# Anticancer Activity of Sea Cucumber Triterpene Glycosides

**DOI:** 10.3390/md13031202

**Published:** 2015-03-06

**Authors:** Dmitry L. Aminin, Ekaterina S. Menchinskaya, Evgeny A. Pisliagin, Alexandra S. Silchenko, Sergey A. Avilov, Vladimir I. Kalinin

**Affiliations:** G.B. Elyakov Pacific Institute of Bioorganic Chemistry, Far-Eastern Branch of the Russian Academy of Science, Prospect 100 letya Vladivostoka, 159, Vladivostok 690022, Russia; E-Mails: d_aminin@hotmail.com (D.L.A.); ekaterinamenchinskaya@gmail.com (E.S.M.); pislyagin@hotmail.com (E.A.P.); sialexandra@mail.ru (A.S.S.); avilov-1957@mail.ru (S.A.A.)

**Keywords:** triterpene glycosides, sea cucumbers, antitumor activities, apoptosis, arrest of cell cycle

## Abstract

Triterpene glycosides are characteristic secondary metabolites of sea cucumbers (*Holothurioidea*, *Echinodermata*). They have hemolytic, cytotoxic, antifungal, and other biological activities caused by membranotropic action. These natural products suppress the proliferation of various human tumor cell lines *in vitro* and, more importantly, intraperitoneal administration in rodents of solutions of some sea cucumber triterpene glycosides significantly reduces both tumor burden and metastasis. The anticancer molecular mechanisms include the induction of tumor cell apoptosis through the activation of intracellular caspase cell death pathways, arrest of the cell cycle at S or G2/M phases, influence on nuclear factors, NF-κB, and up-down regulation of certain cellular receptors and enzymes participating in cancerogenesis, such as EGFR (epidermal growth factor receptor), Akt (protein kinase B), ERK (extracellular signal-regulated kinases), FAK (focal adhesion kinase), MMP-9 (matrix metalloproteinase-9) and others. Administration of some glycosides leads to a reduction of cancer cell adhesion, suppression of cell migration and tube formation in those cells, suppression of angiogenesis, inhibition of cell proliferation, colony formation and tumor invasion. As a result, marked growth inhibition of tumors occurs *in vitro* and *in vivo*. Some holothurian triterpene glycosides have the potential to be used as P-gp mediated MDR reversal agents in combined therapy with standard cytostatics.

## 1. Introduction

Sea cucumbers (or holothurians), belonging to the class Holothuroidea (Echinodermata), are echinoderms phylogenetically related to sea stars, sea urchins and sea lilies. They are habitually found in the benthic areas and deep seas around the world. They have a leathery skin and an elongated body, and many of them are indeed shaped like soft-bodied cucumbers. This class has around 1100 described living species [1]. Some of them are edible and considered as a delicacy in many countries. Consequently, sea cucumbers have some commercial value and are extensively harvested. Sea cucumbers, also called trepang, bêche-de-mer, or balate, have been used as food and Asiatic folk medicine. Ancient Chinese medical manuscripts reveal that parts of holothurians can improve human immune status enforcing resistance to many diseases and even have an anticancer effect. That is probably why the popular Chinese name for sea cucumber is haishen, which means, roughly, “ginseng of the sea” because ginseng, a plant belonging to the family Araliaceae, has similar medicinal properties [2].

On the other hand, many holothurians, particularly tropical species, are toxic. Toxins are elaborated in the body wall and in the skin and may be released into the sea water continuously, or only when the animal is molested [3,4,5,6,7]. Aborigines of Guam and other regions of the Indo-Pacific used some holothurians to poison small lagoons of coral reefs at low tide for killing fish [8].

The low molecular weight compounds, triterpene glycosides, have long been suggested the main poisonous substances of the sea cucumbers and to play a role in the defense of holothuroids as a toxin against predators and pathogens [9,10,11]. The lanostane triterpene glycosides are characteristic of sea cucumbers (Holothurioidea, Echinodermata). The majority of them have 18(20)-lactones in aglycone and belong to the holostane series. Their carbohydrate chains have from two to six monosaccharide residues including glucose, quinovose, xylose, and 3-*O*-methylglucose and sometimes 6-*О*-acetylglucose, 3-*O*-methylxylose, 3-*О*-methylglucuronic acid, and 3-*О*-methylquinovose. Carbohydrate chains may have from one to three sulfate groups [12].

These compounds have a wide range of pharmacological properties. During the last decade, several reviews on the study of the cytotoxic activity of triterpene glycosides have been published. These surveys have shown a correlation between the structure of triterpenoid saponins and its cytotoxic activity related to the molecular mechanisms of action [9,10,11,12]. Most of the glycosides have cytotoxic, hemolytic, antifungal, and similar biological activities caused by membranotropic action at milli- and micromolar concentrations. The membranotropic action of the glycosides is caused by their ability to attach to cell membranes and form nonselective ion-conducting complexes with 5(6)-unsaturated sterol, preferably with cholesterol, followed by an efflux of some ions, nucleotides, and peptides. The following breaking of ion homeostasis and osmolarity results in cell lysis and death [12]. In addition to cytotoxic properties, these glycosides block egg cleavage and development of sea urchin embryos, inhibit the growth of pathogenic fungi and proliferation of some types of human tumor cells *in vitro* such as U-87-MG, HCT-8, leukemia P-388, KB, Schabel, Mel-28, A-549, MICF-1, HT-29, IA9, CAKI-1, SK-MEL, PC-3, lymphoidal leukemia L 1210, MCF-7, MKN-28, HCT-116, U87MG, HepG2, HeLa, THP-1, KB-VIN, HCT-8, C33A, and some others [9,10,11,12,13,14,15,16,17,18,19].

In recent years, holothurian triterpene glycosides have attracted the attention of experimental oncologists as potential anticancer natural compounds. The current review summarizes the recent data on anticancer activity of sea cucumber triterpene glycosides and some aspects of their molecular mechanisms upon cancer cells.

## 2. Anticancer Activity

The first anticancer properties of the sea cucumber glycoside, holothurin, representing the glycoside fraction of Bahamian sea cucumber *Actinopyga agassizi,* were described in 1952 by Nigrelli [20]. He showed that the injection of holothurin, which is a mixture of triterpene glycosides containing as a main constituent holothurin A, in the region of Sarcoma-180, inhibited tumor growth and caused its regression in mice.

Later investigations of holothurin have shown promise in the field of cancer research. Thus, the injection of Krebs-2 ascitic tumor cells treated with holothurin into healthy mice failed to induce marked tumor growth for up to 80 days [21,22]. In addition, holothurin was shown to inhibit the growth of epidermal carcinoma (KB) tumor cells [23,24].

Later, more in-depth studies of the mechanisms of glycoside antitumor action were conducted. Thus, new triterpene glycosides, philinopsides A, B, E and F, as well as pentactasides I, II and III have been isolated from the sea cucumber *Pentacta quadrangularis*. All the glycosides revealed significant cytotoxicities *in vitro* against such tumor cell lines as U87MG, A-549, P-388, MCF-7, HCT-116, and MKN-28 with IC_50_ in the range of 0.60–3.95 µM [13,25].

**Chart 1:**
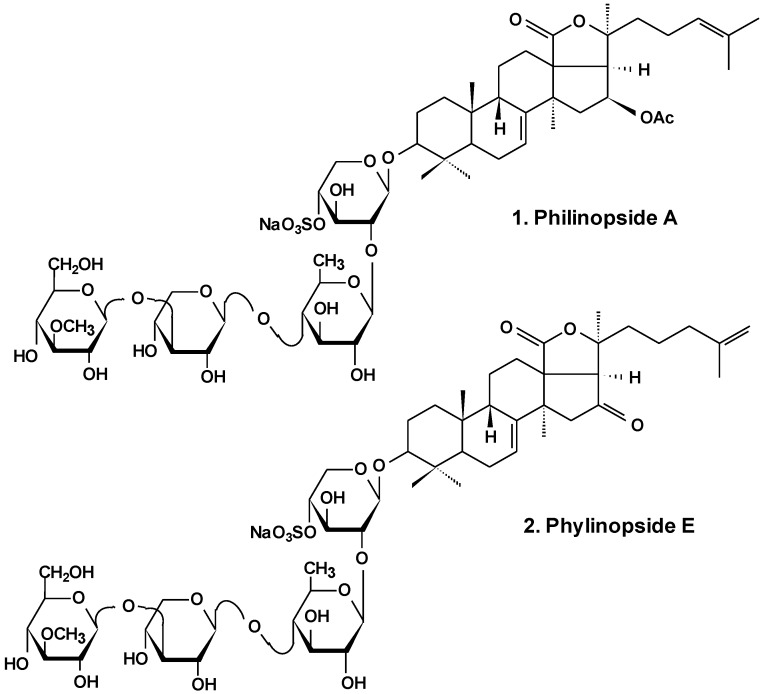
Structure of philinopsides.

In the most extensive research, philinopside A (**1**), one of the potent cytotoxic glycosides (Chart 1), was shown to have effects upon angiogenesis as well as tumor growth. These effects were assessed in a series of models *in vitro* and *in vivo*. Results showed that due to significant inhibition of three important stages of angiogenesis (endothelial cell proliferation, migration, and tube formation) induced by philinopside A, the formation and growth of new blood vessels were greatly decreased. At various doses, philinopside A induced inhibition of proliferation of human microvascular endothelial cells (HMECs) by 98.7%. At the same doses, the glycoside induced the inhibition of HMECs migration by 94.1%. Rat aorta culture assay provides a close imitation of *in vivo* angiogenic processes. In this model, 2–10 μM philinopside A suppressed the formation of new microvessels. Additionally, in the chick embryo chorioallantoic membrane assay, philinopside A, at 2–10 nmol/egg, significantly inhibited angiogenesis. Philinopside A also manifested strong anti-tumor activities both *in vitro* and *in vivo*. The glycoside reduced the volume of mouse Sarcoma-180 tumor by inducing apoptosis of tumor along with tumor-associated endothelial cells. Studies of the action of philinopside A on the angiogenesis-related receptor tyrosine kinases (RTKs) revealed that philinopside A broadly inhibited all tested RTKs, including fibroblast growth factor receptor-1 (FGFR1), platelet-derived growth factor receptor-β (PDGFβ), vascular endothelial growth factor receptor (VEGFR), along with epithelial growth factor receptor (EGFR), at IC_50_ values ranging from 2.6 to 4.9 μM. These results suggest that philinopside A, because of its inhibition of all the tested RTKs, might prove to be an effective inhibitor of RTK, while a lethal dose (LD_50_) in mice was only 625 mg/kg orally [26].

Recently, anti-tumor and anti-angiogenesis activities of philinopside E (**2**), a sulfated saponin from sea cucumber *Pentacta quadrangularis* (Chart 1), were examined. Inhibition of angiogenesis was assessed *in vitro* using proliferation, migration, adhesion, tube-formation and apoptosis assays in philinopside E-treated human dermal microvascular endothelial cells and human umbilical vein endothelial cells. The results showed that philinopside E inhibited proliferation of dermal microvascular endothelial cells and umbilical vein endothelial cells with IC_50_ values of 2.22 ± 0.31 μM and 1.98 ± 0.32 μM, respectively. This glycoside induced the apoptosis of endothelial cells at concentrations <2 μM, dose-dependent suppression of cell migration, adhesion of cells and formation of tubes in those cells, and revealed anti-proliferative activities against a series of tumor cell lines (IC_50_ values of ~4 μM). Philinopside E (5 nM/egg) suppressed spontaneous angiogenesis in the chorioallantoic membrane assay *in vivo* and induced significant inhibition of growth in mouse Hepatoma-22 and Sarcoma-180 cell models. Specifically, philinopside E reduced the tumor volume of mouse Sarcoma-180 by triggering apoptosis of both tumor and tumor-associated endothelial cells, preferentially of endothelial cells over tumor cells. Philinopside E also suppressed the active (phosphorylated) forms of vascular endothelial growth factor receptors including: KDR/Flk-1 (which trigger downstream signaling pathways), VEGF2 ERK (which is required for the mitogenic activities of VEGF in endothelial cells), FAK (which regulates mitogenicity), paxillin (which associates with FAK and plays an important role in cell adhesion and migration and is involved in proliferation, adhesion, migration and survival of endothelial cells), and Akt (which regulates cell survival). These data indicate that philinopside E induces an anti-angiogenic activity associated with inhibition of signaling of VEGFR2, and has pronounced anti-tumor activity caused by the decrease of proliferation of tumor cells and increase of apoptosis of both tumor and endothelial cells [27]. Additionally, it was demonstrated that philinopside E specifically interacts with the extracellular domain of kinase insert domain-containing receptor (KDR) and blocks its interaction with VEGF and its downstream signaling. This specificity for the KDR extracellular domain is distinct from conventional small-molecule inhibitors that target the KDR cytoplasmic domain. It was also shown that philinopside E significantly suppresses α_v_β_3_ integrin-driven downstream signaling caused by a disturbance of the interaction between KDR and α_v_β_3_ integrin in HMECs, followed by a disruption of the cytoskeleton organization of actin and decreased adhesion of cells to vitronectin [28].

**Chart 2:**
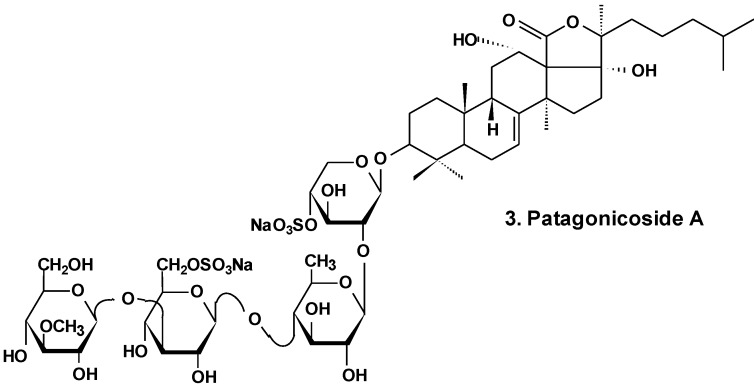
Structure of patagonicoside A.

Patagonicoside A (**3**) from *Psolus patagonicus* (Chart 2) and its desulfated analogs were studied for their cytotoxic, antiproliferative, and hemolytic activities and their influence on NF-κB activation. Both substances were able to suppress the growth of three tumor cell lines (Hep3B, MDA-MB231, and A549) and induced the activation of NF-κB, a key player linking chronic inflammation and cancer, concomitant with IK Ba degradation in the A549 tumor cell line. These compounds showed hemolytic activity with half maximal inhibitory concentration (IC_50_) values around 80 μM. Both glycosides showed low cytotoxic activity in A549 tumor cells in comparison with other sea cucumber triterpene glycosides containing linear tetrasaccharide chains. This probably is because of the presence of an additional sulfate at C-6 of glucose residue (third monosaccharide residue). This also could be a result of the uncommon presence of two 12α- and 17α-hydroxyl groups and a Δ^7^ double bond in the aglycone moiety [29].

**Chart 3:**
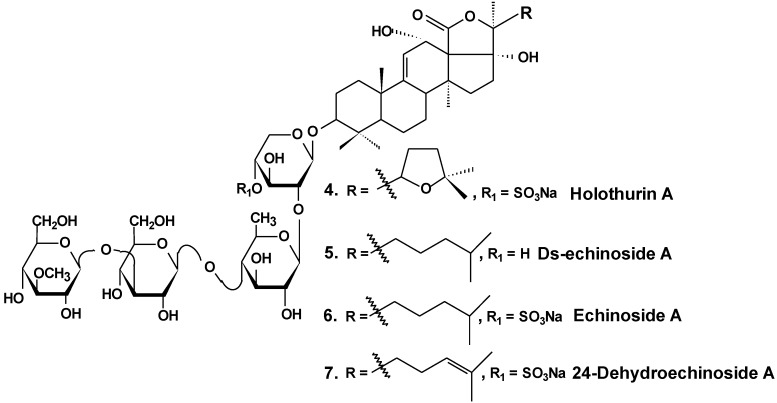
Structures of holothurin A and echinosides.

*In vitro* and *in vivo* investigations were conducted on the effects of Ds-echinoside A (**5**), a non-sulfated triterpene glycoside isolated from the sea cucumber *Pearsonothuria graeffei* (Chart 3), on tumor cell adhesion, migration, invasion, and angiogenesis. In this study, it was found that Ds-echinoside A reduced cell viability of human hepatocellular liver carcinoma cells Hep G2, with IC_50_ of 2.65 μmol/L, and suppressed adhesion, migration, and invasion of Hep G2 cells in a concentration-dependent manner. Ds-echinoside A also decreased tube formation of human endothelial cells ECV-304 on matrigel *in vitro* and attenuated neovascularization in the chick embryo chorioallantoic membrane assay *in vivo*. Immunocytochemical analysis showed that Ds-echinoside A suppressed matrix metalloproteinase-9 (MMP-9) expression, playing an important role in breaking basement membranes associated with angiogenesis and metastasis. Ds-echinoside A also increased the expression of tissue inhibitors of metalloproteinase-1 (TIMP-1) regulating activation of MMP-9. Ds-echinoside A also reduced the expressions of vascular endothelial growth factor (VEGF) and NF-κB [30].

Both echinoside A (**6**) and Ds-echinoside A (**5**) (Chart 3) significantly arrested the cell cycle in the G_0_/G_1_ phase. A reverse transcriptase-polymerase chain reaction assay showed that glycosides increased the cell-cycle-related genes expression, including p16, p21, and c-myc, and decreased the expression of cyclin D_1_. They decreased the expression of Bcl-2, and enhanced mitochondrial cytochrome c release, caspase-3 activation, and cleavage of adenosine diphosphate ribose polymerase. Ds-echinoside A significantly decreased Nuclear factor NF-κB expression, but echinoside A was not involved in concerning the expression. Echinoside A and Ds-echinoside A (2.5 mg/kg) induced the reduction of H22 hepatocarcinoma tumor weight by 49.8% and 55.0%, respectively [31].

Moreover, it was shown that echinoside A reduced tumor growth in mouse and xenografts of human prostate carcinoma in nude mice models. Echinoside A inhibited the noncovalent binding of Top2alpha to DNA by competing with DNA for the DNA-binding domain and by interfering predominantly with the Top2alpha-mediated prestrand passage cleavage/religation equilibrium over with the poststrand passage. These characteristics distinguish echinoside A from known Top2alpha inhibitors. Hence, echinoside A induced DNA double-strand breaks in a Top2-dependent manner [32].

Similar results were obtained by the authors in an earlier study of two sulfated triterpene glycosides, namely, holothurin A (**4**) and 24-dehydroechinoside A (**7**), from the sea cucumber *Pearsonothuria graeffei* (Chart 3). Both of these glycosides exhibited significant inhibition of metastasis *in vitro* and *in vivo*. Immunocytochemical analysis revealed that both compounds significantly decreased the expression of MMP-9 and enhanced the expression level of tissue inhibitors of TIMP-1, an important regulator of MMP-9 activation. According to the results of Western blot analysis, both chemicals remarkably abolished the expression of VEGF. In contrast, the treatment of 24-dehydroechinoside had no effect on the down regulation of NF-κB expression and considerably reduced the adhesion of HepG2 to both matrigel and ECV-304 and also inhibited HepG2 cell migration and invasion in a concentration-dependent manner. 24-dehydroechinoside more effectively induced antimetastasis than holothurin A. Moreover, only holothurin A downregulated the expression of NF-κB. This suggests that antimetastatic activity of the glycosides of *P. graeffei* can be either NF-κB-dependent or -independent, depending on glycoside chemical structure [33].

**Chart 4:**
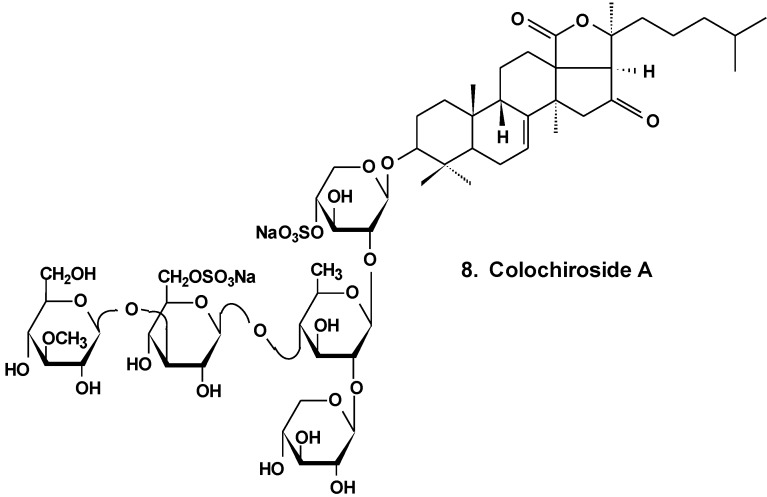
Structure of colohiroside A.

It was found that the colochiroside A (**8**) from the sea cucumber *Colochirus anceps* (Chart 4) remarkably exhibited antineoplastic activities *in vitro* and *in vivo* and did not reduce the immunoregulatory function of mice. The preliminary cytotoxic assay of colochiroside A revealed significant cytotoxic activity against six types of cultured tumor cell lines of P-388, HL60, A-549, SpC-A4, MKN-28, and SGC-7901, with a mean IC_50_ of 3.61 ± 0.55 mg/L. The preliminary anti-tumor assay of colochiroside A suggests that this glycoside exhibits strong inhibitory effects against H22 liver cancer and S180 sarcoma cells in mice. The maximal inhibition ratio to H22 liver cancer was 52.2%, while the ratio to S180 sarcoma was 70.0%. The immunoregulatory study indicated that colochiroside A has no significant effect on the developments of thymus and spleen [34].

**Chart 5:**
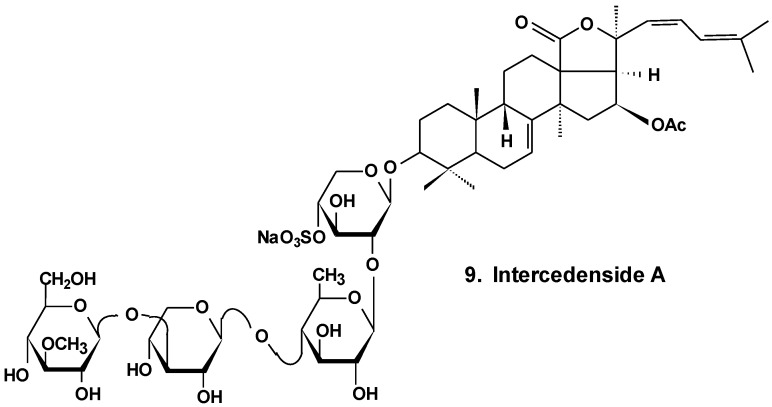
Structure of intercedenside A.

Intercedensides A, B, and C from *Mensamaria intercedens* exhibited cytotoxicity against human tumor cell lines. One of them, intercedenside A (**9**) (Chart 5), also revealed good antineoplastic activity against mouse Lewis lung cancer and mouse S180 sarcoma [35].

**Chart 6:**
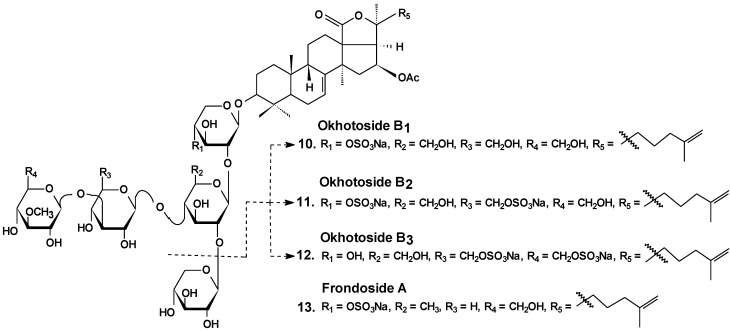
Structures of okhotosides and frondoside A.

Okhotosides B_1_ (**10**), B_2_ (**11**) and B_3_ (**12**) from *Cucumaria okhotensis* (Chart 6) were moderately toxic against cells of HeLa tumor. Frondoside A (**13**) isolated from the same holothurian revealed more potent cytotoxicity against THP-1 and against HeLa tumor cells (with IC_50_ values of 4.5 and 2.1 μg/mL, respectively). This substance decreased both the AP-1-dependent trascriptional activities induced by UVB, EGF, or TPA in JB6-LucAP-1 cells and the EGF-induced NF-κB-dependent transcriptional activity in JB6-LucNF-κB cells at doses of about 1 μg/mL. Frondoside A increased the p53-dependent transcriptional activity in nonactivated JB6-Lucp53 cells at the same doses. It also inhibited the colony formation of JB6 P (+) Cl 41 cells activated with EGF (INCC_50_ = 0.8 μg/mL) [36].

The tumor suppressing and pro-apoptotic activity of the different water-soluble holothurian glycoside fractions from *Apostichopus japonicus* were examined. The 70% ethanol fraction from macroporous resin column and the pSC-2 and pSC-3 fractions from a silica gel column showed very strong tumor suppressing activity towards HeLa cells, A-549 lung cancer cells, SGC-7901 stomach cancer cells, and Bel-7402 liver cancer cells. SC-2 and SC-3 fraction purified by Sephadex LH-20 gel-filtration column chromatography, with purity above 99.6%, all had the properties of triterpenoid glycosides. Purified SC-2 fractions had remarkable tumor suppressing activity on HeLa cells in a dose- and time-dependent manner, and had prominent tumor suppressing activity on mouse S180 solid tumors in a dose-dependent manner. Additionally, the SC-2 fraction also had a remarkable ability to elevate mouse thymus and spleen indexes. The purified SC-2 fraction induced apoptosis of HeLa cells in a dose-dependent manner and DNA fragmentation of HeLa cells occurred after 12 h treatment with 10 mg/L and 50 mg/L of SC-2 fractions [37].

**Chart 7:**
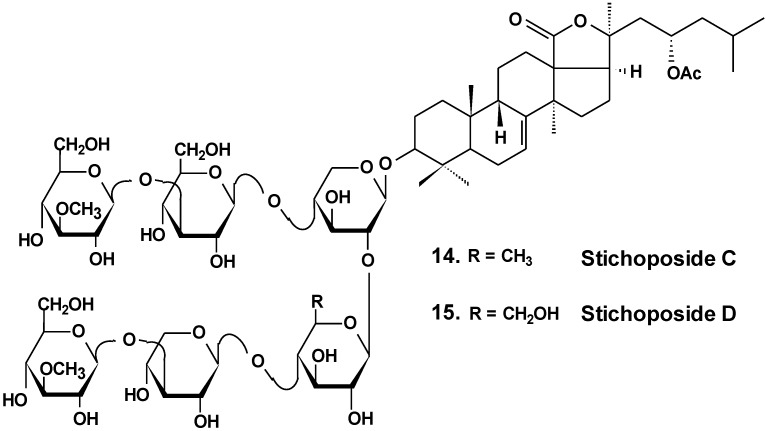
Structure of stichoposides.

Stichoposide C (**14**) from the holothurian *Thelenota anax* (Chart 7) was examined for elucidation of possible mechanisms by which it induces apoptosis of cancer cells. Stichoposide C-induced apoptosis in human leukemia and colorectal cancer cells were examined in the context of mitochondrial injury and signaling pathway disturbances. Additionally, the antitumor effects of stichoposide C in mouse CT-26 subcutaneous tumors and HL-60 leukemia xenograft models were investigated. It was found that stichoposide C induced apoptosis in these cells in a dose-dependent manner leading to the activation of Fas and caspase-8, cleavage of Bid, mitochondrial damage, and caspase-3 activation. Stichoposide C activated neutral SMase (SMase) and acid sphingomyelinase, resulting in ceramide generation. The knockdown experiments concerning specific inhibition of neutral SMase or acid SMase and siRNA partially blocked apoptosis induced by stichoposide C. Moreover, the glycoside significantly decreased growth of HL-60 xenograft tumors and CT-26 subcutaneous tumors and increased generation of ceramide *in vivo*. The authors concluded that ceramide generation by stichoposide C because of the activation of neutral and acid SMase, may contribute to the apoptosis and the antitumor activity induced by stichoposide C [38].

Significant contribution to the understanding of the molecular mechanisms of antitumor actions of triterpene glycosides has been made in the study of frondoside A (**13**) (Chart 6) and its analogs isolated from the sea cucumber *Cucumaria frondosa*. Chemopreventive efficacy of Frondanol A5, a preparation from *C. frondosa* which contains several sea cucumber-derived anticancer and antiinflamatory agents including the triterpene glycoside frondoside A, was evaluated on azoxymethane-induced rat colon carcinogenesis using colonic aberrant crypt foci (ACF) as a surrogate biomarker. Rats were fed the AIN-76A diet containing 5% corn oil, and ACFs were induced by azoxymethane. Three days after azoxymethane treatment, rats were fed with diets containing Frondanol A5. It was shown that dietary administration of 150 and 450 ppm of Frondanol A5 markedly decreased total colonic ACF formation induced by azoxymethane, approximately 34% to 55%, and multicrypt aberrant foci (48%–68.5%) depending on the dosage. ACFs in rats treated with Frondanol A5 revealed marked up-regulation of p21WAF1/CIP1 and down-regulation of proliferating cell nuclear antigen. Frondanol A5 revealed inhibition of the growth at S and G2/M phases along with a decrease in Cdc25c and an increase in p21WAF1/CIP along with significant apoptosis caused by H2AX phosphorylation and the cleavage of caspase-2 in HCT116 cells. The authors concluded that Frondanol A5 exhibits potential chemopreventive properties for colon carcinogenesis [39].

A polar precipitate sub-fraction of Frondanol A5 (Frondanol A5P) was examined for anti-cancer effects in S2013 and AsPC-1 human pancreatic cancer cell lines. Frondanol-A5P inhibited cell proliferation and induced cell cycle arrest at G2/M phase in both cell lines with decreased expression of cdc25c, cyclin A, and cyclin B. Frondanol-A5P also induced phosphorylation of Janus kinase (SAPK/JAK) and stress-activated protein kinase along with p38 mitogen-activated protein kinase (MAP) during 5 min and markedly increased expression of p21waf1 messenger RNA and protein at 3 h in both cell lines. This effect was decreased by the inhibitor of p38 kinase, SB203580. Frondanol-A5P also significantly increased annexin V binding and caspase-3 activation [40].

It was established that individual frondoside A (**13**) from *Cucumaria frondosa* inhibited cell proliferation of AsPC-1 human pancreatic cancer in a concentration- and time-dependent manner. In concert with inhibition of cell growth, frondoside A induced significant morphological changes consistent with apoptosis. Its activity led to an increase of sub-G0/G1 apoptotic cells population, a decrease in expression of Bcl-2 and Mcl-1, an increase in Bax expression, an increase in the expression of the cyclin-dependent kinase inhibitor, p21, and activation of caspases 3, 7, and 9. These data revealed that frondoside A induced apoptosis of human pancreatic cancer cells via the mitochondrial pathway and activation of the caspase cascade. Frondoside A (10 μg/kg/day) inhibited growth of AsPC-1 in xenograft mouse models [41].

The impact of frondoside A on human breast cancer cell line MDA-MB-231 was compared to the effect on a non-tumorigenic MCF10-A cell line derived from normal human mammary epithelium. The glycoside (0.01–5 μM) decreased breast cancer cell viability in a concentration- and time-dependent manner, with EC_50_ of 2.5 μM at 24 h. MCF10-A cells were more resistant to the cytotoxic action (EC_50_ superior to 5 μM at 24 h) [42].

Frondoside A significantly increased sub-G1 (apoptotic) cell fractions by the activation of p53 followed by the appearance of caspases 9 and 3/7 cell death pathways in the MDA-MB-231 cells. Moreover, frondoside A induced inhibition of MDA-MB-231 cell migration and invasion in concentration-dependent manner. Frondoside A, at the dosage of 100 μg/kg/day intraperitoneal for 24 days, effectively decreased the growth of tumor xenografts in athymic mice *in vivo* without toxic-side action. Frondoside A also increased the anti-proliferative activity of paclitaxel in such breast cancer models [42].

It was shown that frondoside A possesses potent antimetastatic activity on syngenic murine model of metastatic breast cancer. Upon intraperitoneal administration to mice with mammary gland-implanted tumors, Frondoside A inhibited spontaneous tumor metastasis in the lungs. The increase of cyclooxygenase-2 activity promotes tumor growth and metastasis by producing high levels of PGE2 that acts on the receptors of prostaglandin E, mainly EP4 and EP2. Frondoside A antagonizes the receptors EP2 and EP4 of prostaglandin E. Frondoside A inhibited ^3^H-PGE_2_ binding to recombinant EP2 or EP4-expressing cells at a high concentration (IC_50_ of 16.5 μM and 3.7 μM, respectively) that may be caused by cytotoxic effects. Moreover, frondoside A also inhibited EP4 or EP2-linked activation of intracellular cAMP along with EP4-mediated ERK1/2 activation. Along with the antimetastatic activity found *in vivo*, frondoside A at concentrations 0.1 and 1.0 μM also inhibited migration of tumor cells *in vitro* in response to EP4 or EP2 agonists [43].

The effects of Frondoside A on the human non-small lung cancer cell LNM35 survival, migration, and invasion *in vitro*, and on tumor growth, angiogenesis, and metastasis alone, and in combination with cisplatin *in vivo* were investigated. Frondoside A induced a concentration-dependent decrease in viability of MCF-7, NCI-H460-Luc2, A549, MDA-MB-435, HepG2, and LNM35 over 24 h along with a caspase 3/7-dependent pathway of cell death. The IC_50_ concentrations of frondoside A at 24 h were 0.7–2.5 μM. Frondoside A also induced an inhibition of the migration of cells, angiogenesis, and invasion *in vitro* in time- and concentration- dependent mode. Frondoside A (0.01 and 1 mg/kg/day intraperitoneal for 25 days) strongly decreased the growth, angiogenesis, and lymph node metastasis of LNM35 tumor xenografts in athymic mice, without toxic side effects. This glycoside in concentrations between 0.1 and 0.5 μM also significantly blocked basal and bFGF induced angiogenesis in the chick embryo chorioallantoic membrane model of angiogenesis assay. Moreover, frondoside A enhanced lung tumor growth inhibition by the chemotherapeutic agent cisplatin [44].

The anti-invasive activity and anti-metastatic effects of this glycoside at non-cytotoxic concentrations against a human breast cancer cell line were investigated along with the inhibitory effect on cell invasion, clonogenicity, and migration in TPA-stimulated cells of human breast cancer. Frondoside A significantly decreased TPA-induced colony formation, migration and invasion in MBA-MB-231 human breast cancer cells. It was shown that MMP-9 induction is very important for the metastasis of different types of cancer tumor cells. It was also found that this glycoside suppresses TPA-induced enzymatic activity of MMP-9, its secretion and expression. This effect was caused by a reduction of the activation of AP-1 and NF-κB. It also correlated with an increase of TIMP-1 and TIMP-2 expression. Frondoside A inhibited the expression of TPA-induced MMP-9, probably because of the suppression of NF-κB and AP-1 signaling pathways. The glycoside decreases the activation of the PI3K/Akt, ERK1/2 and p38 MAPK signals. These data suggest that frondoside A anti-metastatic effects on human breast cancer cells may be caused by the inhibitin of TPA activation of AP-1 and NF-κB and a decrease of TPA activation of ERK1/2, PI3K/Akt and p38 MAPK signals leading to downregulation of the expression of MMP-9. Such results revealed the role of frondoside A in metastasis and its underlying molecular mechanisms. These data also suggest frondoside A may be used as a chemopreventive agent for metastatic breast cancer [45].

**Chart 8:**
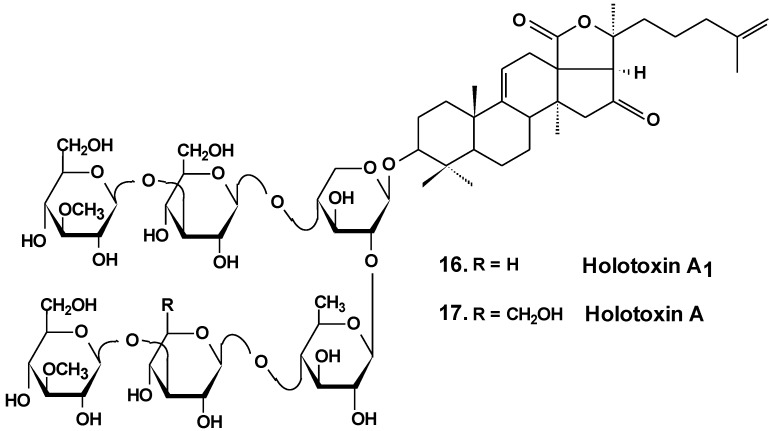
Structures of holotoxins.

**Chart 9:**
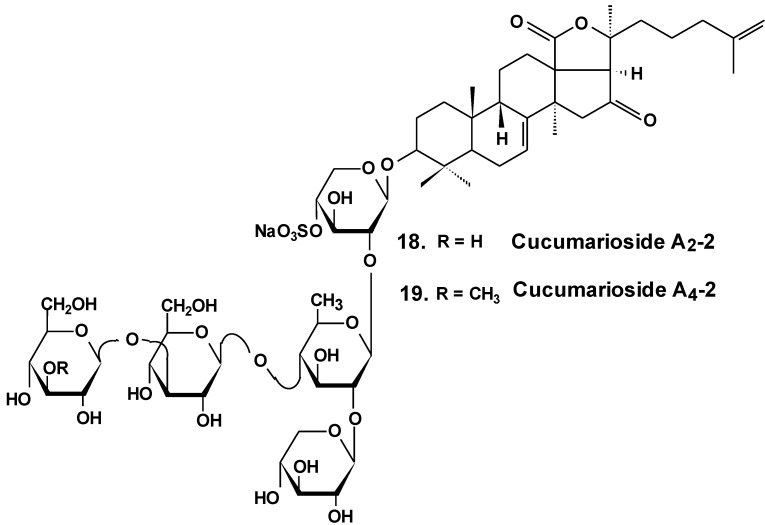
Structures of cucumariosides.

The estrogenic potency of holothurin A (**4**), holotoxin A_1_, (**16**) (Chart 8) frondoside A (**13**), cucumarioside A_2_-2 (**18**) (Chart 9) and a semi-synthesized plant glycoside, ginsenoside-Rh2, were studied using a yeast two-hybrid system including expressed genes of human estrogen receptor, hERalpha, the co-activator TIF2 and lacZ as a reporter gene. It was found that only ginsenoside-Rh2 had moderate estrogenic activity in a concentration range between 10^−7^ and 10^−6^ M. The effect was about 30% of the activity of 17beta-estradiol at half-effective concentration. Hence, ginsenosides-Rh2 is a weak phytoestrogen. Holothurin A, cucumarioside A_2_-2, holotoxin A_1_ and frondoside A did not interact with estrogen receptors and had no appreciable estrogenic activity. These results showed that the anticancer effect of tested holothurian glycosides upon ER-positive breast cancer cells does not involve glycoside binding to estrogen receptors. Ginsenoside-Rh2 has some similarity in chemical structure with 17beta-estradiol. It might explain the affinity of this glycoside to the hER receptors [46].

Frondoside A influenced the anti-cancer effects of gemcitabine, the standard drug in antitumor therapy, against human pancreatic cancer cell lines, AsPC-1 and S2013. The combinations of low concentrations of these preparations were used for a 72 h treatment period *in vitro*. Synergetic growth inhibition was significantly greater than their separate effects. Combinations of gemcitabine and glycosides were tested *in vivo* on the nude mouse model. Xenografts of AsPC-1 and S2013 cells form the tumors before the dministration of the drugs alone or in combination for 30 days. Tumors grew quickly in control mice. The growth was significantly decreased in all treatment groups. Gemcitabine (4 mg/kg/dose), combined with the glycoside (100 μg/kg/day) was more effective than the use of either drug alone. It was suggested that combinations of frondoside A and gemcitabine may provide clinical benefits for patients with pancreatic cancer [47].

Sea cucumber triterpene glycosides with differing chemical structures were examined *in vitro* for effects against leukemia cell lines. Cucumariosides A_2_-2 (**18**) and A_4_-2 (**19**) from *Cucumaria japonica* (Chart 9) and stichoposides С (**14**) and D (**15**) from *Thelenota anax,* in cytotoxic doses, were shown to induce apoptosis in human leukemia cells HL-60, THP-1, NB-4 and K562 *in vitro* by a caspase-dependent mechanism. Thus, sea cucumber triterpene glycosides, in spite of differing molecular structures, may nonetheless have generalized therapeutic relevance for human cancer [48].

The effects of frondoside A from *C. frondosa,* cucumarioside A_2_-2, and cucumarioside A_4_-2 from *C. japonica* on cell death-inducing capability were compared. These glycosides significantly induced apoptosis of leukemic cells. The apoptosis induced by frondoside A was more potent and rapid than apoptosis induced by cucumarioside A_2_-2. Mitohondrial membrane permeability was not changed and accumulation of cytochrome C in the cytosolic fraction was not found in HL-60 cells treated with frondoside A, cucumarioside A_2_-2 and cucumarioside A_4_-2. More interestingly, the level of procaspase-3, -8, and -9 proteins in lysates from frondoside A-treated HL-60 cells were not changed, whereas frondoside A-induced apoptosis in 50%–70% of the cells. Cleavage of procaspase-3 and PARP, but not of procaspase-8, -9, and -12, were significantly increased in cucumarioside A_2_-2 or cucumarioside A_4_-2 treated HL-60 cells. Furthermore, the annexin-V positivity in cells treated by fronfdoside A was not inhibited by zVAD-fmk. Nevertheless, both annexin-V positivity and cleavage of caspases induced by cucumariosides were efficiently suppressed by caspase inhibitors. This suggests that holothurian triterpene glycosides may induce apoptosis of leukemic cells caspase-dependently or independently, depending on the glycoside structure [49].

In another investigation, the cytotoxicity of cucumarioside A_2_-2 and its effect upon apoptosis, the cell cycle, DNA biosynthesis and p53 activity, and glycoside anticancer action against mouse Ehrlich carcinoma cells were studied. It was found that the glycoside influences viability of tumor cells at micromolar concentrations. The EC_50_ for glycoside found by non specific esterase assay and MTT assay was 2.1 and 2.7 μM, respectively. The glycoside at sub-cytotoxic range of concentrations revealed a cytostatic effect by blocking the proliferation of cells and biosynthesis of DNA in the S phase. Cucumarioside A_2_-2 may induce apoptosis in cells of Ehrlich carcinoma along with caspase-dependent ways by passing activation of p53-dependent segments. It was concluded that the anticancer and pro-apoptotic properties of the glycoside may be caused by interaction of cucumarioside A_2_-2 with tumor cells. The anticancer effect of cucumarioside A_2_-2 *in vivo* may be caused by the ability of the substance to arrest the cell cycle in the DNA synthetic phase and induce programmed death of tumor cells [50].

Additionally, it was found that in non-cytotoxic concentrations of frondoside A and cucumarioside A_2_-2, as well as their complexes with cholesterol, block the activity of membrane transport P-glycoprotein. This protein is responsible for multidrug resistance (MDR) phenomena in cells of the ascite form of mouse Ehrlich carcinoma. In this way, glycosides prevent an efflux of fluorescent probe Calcein from the cells. Cucumarioside A_2_-2 was found to increase the upload and intracellular concentration of cytostatic doxorubicine, and prevent an efflux of anticancer drugs from the cancer cells. Because the interaction of the glycosides with tumor cells resulted in a decrease of MDR, these glycosides are potential inhibitors of multidrug resistance and can be used in combined therapy of cancer [51,52,53].

As a result of long-term investigations, a new immunomodulatory lead Cumaside with antitumor properties was invented on the base of triterpene glycosides isolated from Far Eastern sea cucumber *Cucumaria japonica*. Cumaside is a complex of monosulfated glycosides (preferably cucumarioside A_2_-2) with cholesterol in molar ratio of 1:2 [54]. Recently, it was found that Cumaside possesses less cytotoxic action against sea urchin embryos and cells of Ehrlich carcinoma than the glycosides. Furthermore, Cumaside posessed antitumor activity against experimental mouse Ehrlich carcinoma *in vivo* both alone and in combination with cytostatics. The highest result was achieved at a treatment of once per day for seven days before the tumor inoculation, followed by Cumaside treatment once per day for seven days. The treatment along with prophylactic schemes with Cumaside and subsequent therapeutic application of 5-fluorouracil inhibited tumor growth by 43% [55].

**Chart 10:**
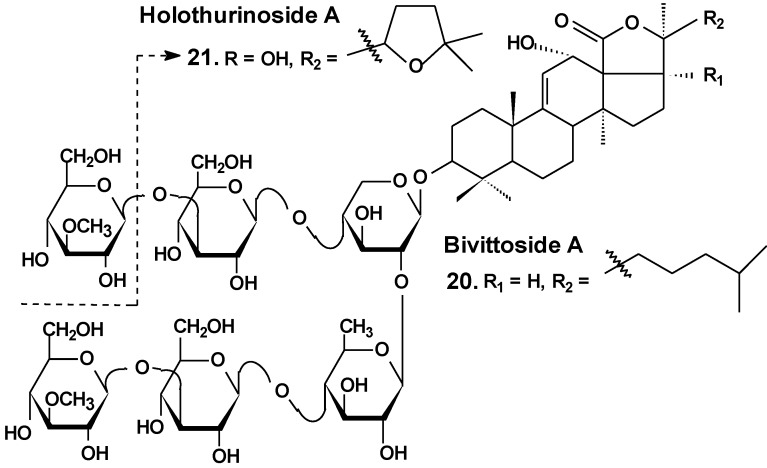
Structure of bivittoside A and holothurinoside A.

Recently, the ability of a number of cytotoxic triterpene glycosides from sea cucumbers to interact with human topoisomerase II alpha (which plays a key role in DNA replication and is a target for a variety of chemotherapeutic agents) has been investigated *in silico* using the methods of computer simulation. This study revealed possible anticancer effects of a series of triterpen glycosides including bivittoside A (**20**), holothurin A (**4**), holotoxin A (**17**), holothurinoside A (**21**) (Chart 10), and cucumarioside A_2_-2 (**18**) using homology modeling of human DNA topo II α. The authors have found the possible binding site of DNA binding domain of Topo II α. These glycosides were screened for QSAR and ADME/TOX analysis as ligands. All the glycosides were able to bind with the enzyme. Binding sites have been established for all the glycosides. All the tested glycosides had a model of antitumor effects. According to this study, cucumarioside A_2_-2 may be a better inhibitor of topo II α and follow most of the ADME properties [56]. The authors explain the inhibition by the interaction of “phenolic principals” of the glycosides with amino acids of the active center of the enzyme by hydrogen bonds. This seems to be an erroneous explanation because the glycosides do not contain phenolic groups. It is possible that the authors have confused pyranose forms of monosaccharide residues with phenols.

A brief amount of information concerning the molecular mechanisms of the biological action of certain sea cucumber triterpene glycosides was collected recently in a review [57]. This review highlights the structural characteristics and mechanisms of the action of marine triterpene glycosides, such as stichoposides, frondoside A, and cucumariosides. In particular, the membranotropic and membranolytic activities of glycosides from sea cucumbers and their ability to induce cytotoxicity and apoptosis had been discussed, with a focus on structure-activity relationships. Membrane transporters, which are modulated by triterpene glycosides and thus can be proposed as potential therapeutic targets, were summarized. These include Na^+^-K^+^-ATPase and Ca^2+^-ATPase in the sarcoplasmic/endoplasmic reticulum, L-type voltage-gated calcium channels, transient receptor potential canonical (TRPC) channels, ryanodine receptor, voltage-gated Na^+^ channels (NaV1.2 and NaV1.4), K^+^ channel (KV1.4), calcium-activated K^+^ channel (BKCa), human Ether-à-go-go Related Gene (hERG), K^+^ channels (Kv11.1), *N*-methyl-d-aspartate (NMDA) receptors and nicotinic acetylcholine receptors, as well as γ-amino butyric acid (GABA) receptors. In addition, the structural characteristics and antitumor effects of some sea cucumber glycosides have been reviewed along with underlying their molecular mechanisms.

Moreover, in recent review, the mechanisms of the anticancer effects of triterpene glycosides, frondoside A and cucumarioside A_2_-2, were summarized with specific emphasis on the apoptotic activity of the glycosides and its effect on metastasis and invasion of cancer cells [58]. The authors concluded that these glycosides may be considered to be both anticancer and cancer preventive agents. Frondoside A and cucumarioside A_2_-2 both posses anti-leukemic properties by inducing apoptosis.

Moreover, the anticancer effects of frondoside A and cucumarioside A_2_-2 might be through inhibition of tumorigenesis and metastasis. The mechanisms are not clear and may be found in the future. Frondoside A will be useful as a caspase-independent anti-leukemic agent in order to overcome chemoresistance associated with a defect in both the extrinsic and intrinsic apoptotic pathways. As a next step, the determination of the structural characteristics responsible for the *in vivo* anticancer activities will be essential for their use as a drug. In addition, oral administration of frondoside A and cucumariosides was recommended by authors in clinical trials.

## 3. Conclusions

In the last few decades, scientific literature from several countries has reported that, indeed, triterpene glycosides from sea cucumbers do have a wide spectrum of biological effects including cytotoxic, hemolytic, antifungal, ichthiotoxic, and other activities. It has been shown that a majority of these activities are based on the interaction of these compounds with membrane sterols. Most triterpene glycosides of sea cucumbers have strong membranotropic action against any cellular and model membranes containing Δ^5^-sterols. This interaction with biomembrane Δ^5^-sterols results in pore formation, changes in membrane viscosity and ion permeability, inhibition of some membrane enzymes (such as ATPases) and others that lead finally to death of the cells.

Simultaneously, some sea cucumber triterpene glycosides exhibit pronounced anticancer effects by direct interaction with tumor cells in the sub-cytotoxic range of concentration. The summarized data of sea cucumber triterpene glycoside effects upon cancer cell viability, cell cycle/proliferation, apoptosis, migration/metastasis, angiogenesis and tumor growth *in vivo* are presented in the Table 1.

**Table 1:** Effects of sea cucumber triterpene glycosides upon cancer cells and tumors*.*

#	Glycoside	Sea Cucumber sp.	Type of Activity
**1**	Philinopside A	*Pentacta quadrangularis*	Reduction of cell viability [13,25], induction of apoptosis, inhibition of angiogenesis and tumor growth *in vitro* and *in vivo* [26,27,28]
**2**	Philinopside E		
**3**	Patagonicoside A	*Psolus patagonicus*	Suppression of cell proliferation [29]
**4**	Holothurin A	*Pearsonothuria graeffei*	Reduction of cell viability [30]; inhibition of cell adhesion, migration, metastasis and invasion [30,33]; induction of apoptosis [31]; cell cycle arrest, reduction of tumor growth *in vivo* [31,32]
**5**	Ds-echinoside A		
**6**	Echinoside A		
**7**	24-dehydro echinoside A		
**8**	Colochiroside A	*Colochirus anceps*	Reduction of cell viability, reduction of tumor growth *in vivo* [34]
**9**	Intercedenside A	*Mensamaria intercedens*	Reduction of cell viability, reduction of tumor growth* in vivo* [35]
**10**	Okhotoside B_1_	*Cucumaria okhotensis*	Reduction of cell viability [36]
**11**	Okhotoside B_2_		
**12**	Okhotoside B_3_		
**13**	Frondoside A	*Cucumaria frondosa* *Cucumaria okhotensis*	Reduction of cell viability [36]; inhibition of colony formation [36,39,45] and cell proliferation [41,42], cell cycle arrest [39,40,41], induction of apoptosis [40,41,42,58]; inhibition of cell migration [43,44,45] and invasion [44,45]; inhibition of metastasis [43,44,58], angiogenesis [44]; inhibition of MDR [51,52,53]; reduction of tumor growth *in vivo* [41,44]; enhancement of tumor growth inhibition by cytostatics [42,44,47]
**14**	Stichoposide C	*Thelenota anax*	Induction of apoptosis; reduction of tumor growth* in vivo* [38]
**18**	Cucumariosides A_2_-2	*Cucumaria japonica*	Inhibition of cell proliferation, cell cycle arrest and induction of apoptosis [48,49,50]; inhibition of metastasis and invasion [58]; reduction of tumor growth *in vivo* [48,49]; enhancement of tumor growth inhibition by cytostatics [55] inhibition of MDR [51,52,53]
**19**	Cucumarioside A_4_-2		

Basically, the detailed mechanism(s) of the anticancer activities of these glycosides still remain largely unclear. However, the general details of this mechanism may be reduced to the following points:

(a) induction of tumor cell apoptosis was shown to be one of the primary causative factors through the activation of intracellular caspase cell death pathways (caspases 3/7 and 9);

(b) arrest of the cell cycle at S or G2/M phases and increase of the sub-G_0_/G_1_ cell population which leads to the block of proliferation and apoptosis;

(c) regulation (up or down) of nuclear factor NF-κB, a key player linking chronic inflammation and cancer;

(d) regulation (up or down) of certain cellular receptors and enzymes participating in cancerogenesis, such as: EGFR (epithelial growth factor receptor, mutations affecting EGFR expression or activity could result in cancer); AKt (or protein kinase B, involved in cellular survival pathways by inhibiting apoptotic processes); ERK (extracellular signal-regulated kinase controlling many cellular processes such as survival, proliferation, differentiation and motility); FAK (focal adhesion kinase, a cell growth, cell proliferation, cell survival and cell migration mediator, often dysfunctional in cells of cancer); and MMP-9 (matrix metalloproteinase-9, implicated in tumor metastasis) and some others.

Finally, administration of some sea cucumber triterpene glycosides leads to reduction in cancer cell adhesion, suppression of cell migration and tube formation in those cells, suppression of angiogenesis, inhibition of cell proliferation, colony formation and tumor invasion, and, as a result, marked growth inhibition of tumors *in vitro* and *in vivo*. Additionally, some holothurians’ triterpene glycosides have the potential to be used as P-gp mediated MDR reversal agents in combined anticancer therapy with standard cytostatics.
